# Angiogenic Signalling Pathways Altered in Gliomas: Selection Mechanisms for More Aggressive Neoplastic Subpopulations with Invasive Phenotype

**DOI:** 10.1155/2012/597915

**Published:** 2012-07-17

**Authors:** Susana Bulnes, Harkaitz Bengoetxea, Naiara Ortuzar, Enrike G. Argandoña, Álvaro Garcia-Blanco, Irantzu Rico-Barrio, José V. Lafuente

**Affiliations:** ^1^Laboratory of Clinical and Experimental Neuroscience (LaNCE), Department of Nursing I, University of the Basque Country, 48940 Leioa, Spain; ^2^Laboratory of Clinical and Experimental Neuroscience (LaNCE), Department of Neuroscience, University of the Basque Country, Leioa, P.O. Box 699, 48080 Bilbao, Spain; ^3^Unit of Anatomy, Department of Medicine, University of Fribourg, 1700 Fribourg, Switzerland

## Abstract

The angiogenesis process is a key event for glioma survival, malignancy and growth. The start of angiogenesis is mediated by a cascade of intratumoural events: alteration of the microvasculature network; a hypoxic microenvironment; adaptation of neoplastic cells and synthesis of pro-angiogenic factors. Due to a chaotic blood flow, a consequence of an aberrant microvasculature, tissue hypoxia phenomena are induced. Hypoxia inducible factor 1 is a major regulator in glioma invasiveness and angiogenesis. Clones of neoplastic cells with stem cell characteristics are selected by HIF-1. These cells, called “glioma stem cells” induce the synthesis of vascular endothelial growth factor. This factor is a pivotal mediator of angiogenesis. To elucidate the role of these angiogenic mediators during glioma growth, we have used a rat endogenous glioma model. Gliomas induced by prenatal ENU administration allowed us to study angiogenic events from early to advanced tumour stages. Events such as microvascular aberrations, hypoxia, GSC selection and VEGF synthesis may be studied in depth. Our data showed that for the treatment of gliomas, developing anti-angiogenic therapies could be aimed at GSCs, HIF-1 or VEGF. The ENU-glioma model can be considered to be a useful option to check novel designs of these treatment strategies.

## 1. Introduction

 Gliomas are the most common type of primary tumour in the central nervous system. Glioblastomas (GBM) are the malignant form of gliomas (World Health Organization Grade IV), and progress from a lower-grade glioma (secondary GBM) or appear *de novo* without any preceding tumour (primary GBM). Glioblastomas are strongly angiogenic tumours displaying a high degree of vascular proliferation and endothelial hyperplasia. It is a neoplasia of glial lineage with high proliferative and invasive capacity and may spread to occupy an entire lobe or even a hemisphere of the brain [1].

The growth of the glioblastoma is related to vascular network adaptation due to the increase of the metabolic necessities of neoplastic cells. During the early stages of gliomas, the metabolic demand is supplied by the vast microvasculature of the CNS; however, when the metabolic supply capacity is exceeded during neoplastic progression, new formation of vessels becomes necessary [2–4]. The genesis of new vessels from preexisting ones is called angiogenesis, in opposition to vasculogenesis, which refers to the formation of vessels from hematopoietic niches [5, 6].

Angiogenesis is a complex phenomenon, necessary for the progression of malignant gliomas [7]. The start of the angiogenesis process requires some angiogenic exogenous stimulus, such as hypoxia, current metabolic requirements, and tumour growth. More than 25 different growth factors and cytokines related to the induction of angiogenesis have been reported [8]. The production of these proangiogenic factors is a result of genetic alterations or is induced by hypoxia.

Intratumour hypoxia occurs at the time when there is an imbalance between oxygen supply and demand, due to irregular and chaotic blood flow [9]. This situation triggers the synthesis of proangiogenic factors such as matrix metalloprotease (MMP-2), angiopoietin-1, phosphoglycerate kinase (PGK), erythropoietin (EPO), and vascular endothelial growth factor (VEGF)-A [10]. Some of these are mediated by the hypoxia inducible factor (HIF-1). The increase of pro-angiogenic factors induces the start of angiogenesis, also known as the “angiogenic switch.” This is a key moment for tumour malignancy, a critical step for the formation of new blood vessels and for the adaptation of the microenvironment to the growth of gliomas [11–13].

Vascular endothelial growth factor (VEGF) is the major regulator of angiogenesis in development [14–16] and disease [17–19]. VEGF-A triggers the angiogenic switch [4], reaching the target cells (endothelial cells) that express VEGF receptors [20]. However, the role of VEGF in nervous tissue is even more extensive. Previous studies have shown that VEGF also has strong neuroprotective, neurotrophic and neurogenic properties [11, 21–23]. The synthesis of VEGF is mediated by hypoxia inducible factor (HIF-1). Recently, researchers have reported that some neoplastic cells with stem cell characteristics called “glioma stem cells” play a pivotal role, inducing angiogenesis via HIF-1/VEGF [24]. Hypoxia has been related to clone selection of tumour cells. Clones adapted to the tumour microenvironment have acquired the phenotype of tumour stem cells, with increased proliferative and infiltrative capacity [25, 26]. Invasion of adjacent normal parenchyma has also been attributed to glioma stem cells.

The onset of angiogenesis is mediated by a cascade of intratumoural events: alteration of the microvasculature network; a hypoxic microenvironment; adaptation of neoplastic cells; synthesis of proangiogenic factors,; finally the generation of new vessels. This chronological sequence of events is difficult to study during human glioma development. In order to follow the natural development of glioma pathogenesis, an endogenous glioma in rats was generated by transplacental administration of the carcinogen ethylnitrosourea (ENU) [27–30]. It has been reported that ENU gliomas are a representative model for human gliomas due to their location and also to their similar molecular and genetic alterations [31]. According to our experience, ENU-gliomas have proven to be a useful tool in the study of many aspects of tumourigenesis and neoangiogenesis. This model permits various neoplastic stages to be isolated, to study the microvascular aberration process, the role of the proangiogenic cytokine VEGF and the glioma stem cells. This knowledge will contribute to define modern targets for anti-tumoural therapies.

## 2. Vascular Endothelial Growth Factor (VEGF)

Angiogenesis is a complex process that requires proteolytic and mitogenic activity of endothelial cells and interaction of these with the extracellular matrix molecules and peri-endothelial support cells (pericytes and smooth muscle cells). Numerous molecules and pathways are involved in this process, such as HIF-1, VEGF-A, its receptors VEGFR-1 and VEGFR-2, the endothelial receptor tyrosine kinase tie-1 and tie-2, and the angiopoietin ligands 1 and 2. Many other molecules, such as PDGF and TGF-B, integrin receptors, and so forth play a very important role.

The VEGF family consists of seven different homologous factors, VEGF-A, VEGF-B, VEGF-C, VEGF-D, VEGF-E, VEGF-F, and placental growth factor (PIGF) [32]. VEGF-A (VEGF) is the predominant form and is a hypoxia-inducible 45 KDa homodimeric glycoprotein. It has five different isoforms, VEGF_123_, VEGF_145_, VEGF_165_, VEGF_189_ and VEGF_206_, that are produced by alternative splicing. Expression of VEGF-A was initially detected in a variety of tumour cell lines, while its receptors VEGFR-1 and VEGFR-2 were predominantly expressed in endothelial cells [33].

VEGF-A acts as a mitogenic, survival, and antiapoptotic factor for the endothelial cells from arteries, veins, and lymph vessels. Faced with increased secretion of VEGF-A and its binding to receptors on the surface of endothelial cells, VEGF-A is a signal transducer leading the production of molecules that include enzymes for the degradation of the extracellular matrix and for increase vascular permeability.

 VEGF-A stimulates endothelial cell (EC) migration, proliferation, survival, permeability and lumen formation. Dvorak [34] described it as vascular permeability factor (VPF) due to its ability to induce leakage through the blood brain barrier (BBB) in some pathological situations [35, 36]. Helmlinger et al. [37] stated that in the vasodilation process, VEGF induced the elongation of endothelial cells but not their proliferation. In the angiogenesis process, VEGF works alongside other factors such as angiopoietin and ephrins [38].

The synthesis of this proangiogenic cytokine has been described in neurons, astrocytes, pericytes, smooth muscle cells, macrophages, lymphoid cells, platelets, and fibroblasts [39]. In tumours, its synthesis is associated with neoplastic and endothelial cells of aberrant microvessels [17]. The VEGF-A gene is hypoxia regulated, due to a binding site for HIF in the promoter [40]. The rapid proliferation of the tumour, accompanied by a poor blood flow, leads to a relatively hypoxic environment in different areas of the tumour [40], resulting in upregulation of VEGF-A.

 VEGF-A expression has been profusely reported in samples obtained from human gliomas [3]. In human glioma biopsies, it has been shown that VEGF-A overexpression correlates directly to proliferation, vascularization, and degree of malignancy, and therefore corresponds inversely to prognosis [18, 19, 41]. The material studied from human biopsies corresponds to advanced stages of tumour development with an adapted microvascular network. However, little is known about VEGF-A expression during glioma progression, especially during the early stages.

## 3. Hypoxia

During glioma progression, microvessels acquire aberrant morphologies, becoming tortuous, irregular and dilated, and displaying vascular leakage [42, 43]. Consequently, the regional blood flow is irregular and chaotic and the functional deficit of blood perfusion induces intratumour hypoxia and ischemia [43]. Recent evidence suggests that a vascular collapse may precede angiogenesis in the development of the glioma. The vascular collapse leads to the death of neighbouring tumour cells and the formation of necrotic areas. In these regions, tissue hypoxia triggers the expression of HIF-1 and consequently VEGF-A, which initiates angiogenesis [44, 45].

Hypoxia is a critical aspect of the glioma microenvironment [46], and it has been associated with poor prognosis, increased angiogenesis, tumour growth, radio-and chemotherapy resistance, and tumour invasiveness [47, 48]. It was observed that HIF-1 was involved in the oxygen-dependent expression of many genes, including those for pro-angiogenic and vascular permeability factors such us: VEGF [12, 13, 49], endothelial nitric oxide synthase (eNOS), angiopoietin, ephrin [45, 50], and others such as: glycolytic enzymes, glucose transporter-1 (Glut-1), transferrin, inducible nitric oxide synthase and heme oxigenase-1 [51], and TWIST [52]. In addition to oxygen levels, HIF-1 expression can be affected by several mechanisms, including the activation of oncogenes such as EGFR or loss of tumour suppressors, like p53 or PTEN [40].

 HIF-1 is a heterodimeric transcriptional factor composed of two subunits, HIF-1*α* and HIF-1*β*. The HIF-1*β* subunit is constitutively expressed, whereas the HIF-1*α* subunit is regulated by oxygen. The subunit HIF-1*α* was originally discovered in 1988 as a unifying factor to the 3′ hypoxia-inducible gene for erythropoietin (EPO). There are three isoforms of the alpha subunit: HIF1*α*, HIF2*α* (also known as endothelial PAS-domain protein 1, EPAS1), and HIF3*α* [53]. HIF1*α* is responsible for the majority of the hypoxia response in cancer. Recently, a link has been discovered between HIF-1*α*, tumour apoptosis, and the necrosis process [54].

## 4. Glioma Stem Cells

It has been reported that the hypoxic microenvironment plays a fundamental role in the induction of the neoplastic phenotype [55–57]. Some studies elucidated that HIF-2*α* was only significantly present in the cancer stem cells, whereas HIF-1*α* was present in both stem and nonstem tumour cells [56, 57].

 Recently, a relationship between HIF-1*α*, tumour migration and stem cell biology has been found [47, 58]. Furthermore, studies of human GBM have described the relationship between the intratumour oxygen gradient and the appearance of tumour stem cells [59]. These cells, called “glioma stem cells” (GSCs), are thought to be responsible for the process of infiltration and subsequent tumour recurrence. Moreover, it has been reported that the relationship between angiogenesis and tumour invasiveness is due to this neoplastic subpopulation. Recently, researchers have reported that GSCs play a pivotal role in inducing angiogenesis via HIF-1/VEGF [24, 60].

Due to the regulatory role of GSCs in tumour angiogenesis, they are currently being considered as a potential antiangiogenic target. It is a fact that nowadays, research in novel antitumour therapies has centred on identifying these neoplastic cells. So far, markers have been used: CD133/Promonin-1, a prominin family of pentaspan membrane proteins; Nestin, a protein found in neural stem cells in SVZ and other markers of neuroepithelial stem cells: Musashi-1, Sox-2, GFAP, Map-2, Neural-tubulin, Neurofilament O4, Noggin, and CD15 have all been proposed in order to identify tumour stem cells [61–63].

In human glioblastoma, GSCs were identified by CD133 expression and associated with bad prognosis of the tumour [64, 65]. However, little is known about their genesis during glioma progression, especially during the early stages.

## 5. An Experimental Model of Endogenous Glioma

As has previously been described, the onset of angiogenesis is mediated by a cascade of intratumoural events, where both tumour and “non-tumour” cells as well as numerous cytokines are involved. In order to follow the angiogenesis process during the neoplasia genesis, we have used an endogenous glioma model. It was generated by transplacental administration of ethylnitrosourea (ENU) in rats [28, 66, 67].

Previous studies have reported that administration of ENU to adult rodents did not induce CNS tumours, while transplacental administration during the perinatal period (last week of gestation and first week of postnatal time) induced tumours in more than half of the offspring [68]. This model has allowed the study of some aspects related to tumour behaviour or possible treatment strategies *in vivo* [69].

This model shows that after the exposure of pregnant Sprague Dawley rats on the 15th day of gestation to a single intraperitoneal injection of 80 mg ENU/kg body weight, more than half of the offspring developed intracerebral tumours, diagnosed as gliomas [17, 27]. According to histopathological features, immunophenotyping, proliferation index for Ki-67, and MRI study, we have demonstrated that this model reproduces quite closely the neuropathology of oligodendrogliomas and glioblastoma [31, 70].

**Figure 1:**
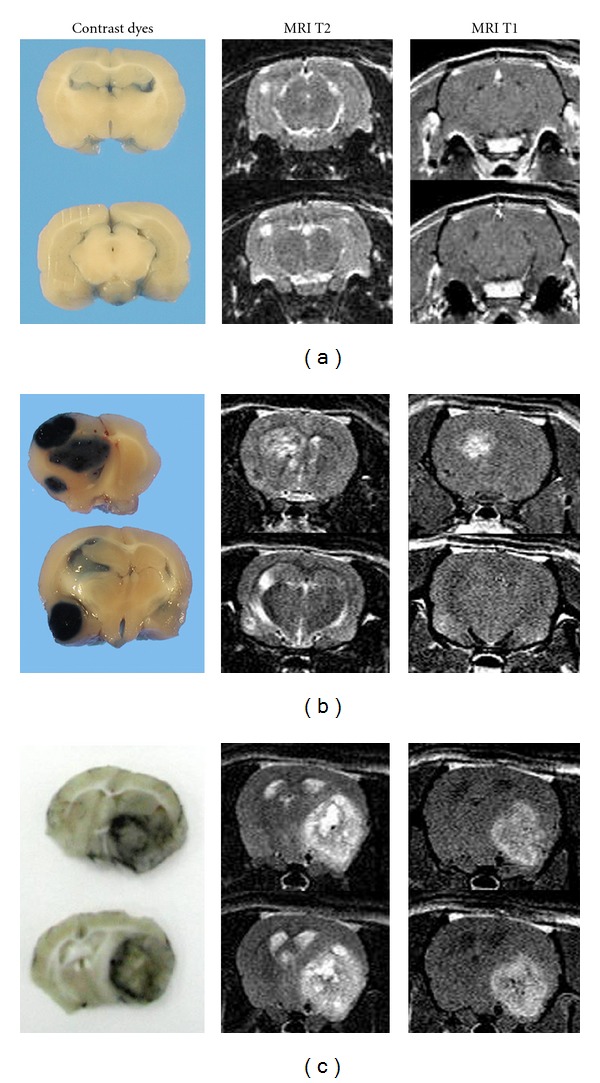
Brain coronal sections of Sprague Dawley rats exposed prenatally to ethylnitrosourea. Two columns show MRI on T1-w and T2-w after gadolinium administration and the left column shows the necropsy thirty minutes after Evans Blue i.v. injection (a, b) or Indian ink (c). (a) On T2-weighted images, tumours in the early development stage show a diminutive proliferation mass growing in association with the subcortical white matter. The blood brain barrier (BBB) remains still intact, shown by the lack of contrast or dye extravasation (microtumours). (b) Multiple tumours on the intermediate development stage. There is a BBB dysfunction indicated by extravasation of Evans Blue and by gadolinium enhancing the contrast on T1 images (macrotumours). (c) A macrotumour in the advanced stage growing over a whole hemisphere. It displays a heterogeneous signal on T2 and on T1 due to the presence of histopathological features of malignancy such as haemorrhages, cysts, and necrosis. With Indian ink, brains show in black a ring of aberrant vessels surrounding the neoplasia. The same typical shape of glioblastoma multiforme may be observed on MRI in T1 with gadolinium.

Three glioma development stages were identified: early (microtumours), intermediate (the smallest macrotumours), and advanced stages (corresponding to the largest macrotumours) (Figure 1). Microtumours corresponding with cell proliferation mass and minor tumour node (Figure 1(a)) were diagnosed as low-degree gliomas, and macrotumours as anaplastic gliomas or high-degree gliomas (Figure 1(b)). The largest macrotumours corresponded to highly infiltrative malignant gliomas (Figure 1(c)). They displayed characteristic features of glioblastomas, such as haemorrhages, microvascular proliferation, macrocysts and necrosis with pseudo-palisades [1, 17]. These results corroborate the origin of secondary GBM from oligodendrogliomas, and not only from astrocytoma cells.

ENU-tumour development shows that malignancy degree is related to rat age and consequently to the glioma size. In a fashion similar to human glioma pathology, the neoplasm has to reach a symptomatic size. At the age of four months, proliferations of oligodendrocytes in the subcortical white matter are frequently seen. These proliferations become nodular at six months, although the rats did not display symptoms. These nodes grow and in one year may occupy a half hemisphere, or extend even more toward the contralateral hemisphere. These macrotumours may display clinical signs, depending on size and malignancy.

Small tumours, characteristic of the early phase, remain asymptomatic and undetected due to their small size, therefore, they are named microtumours. Several authors have considered these noninvasive tumours as dormant tumours. The existence of dormant tumours has notable implications for the early detection and treatment of cancer [71].

## 6. Glioma Hypoxic Microenvironment

In primary tumours, the dormancy state is best defined as the time between carcinogenic transformation and the onset of inexorable progressive growth. There are several mechanisms related to the emergence from tumour latency, and one of them is the induction of angiogenesis. The escape of tumours from dormancy depends on the cell population undergoing an angiogenic switch that induces the neoformation of functional vessels [72].

**Figure 2:**
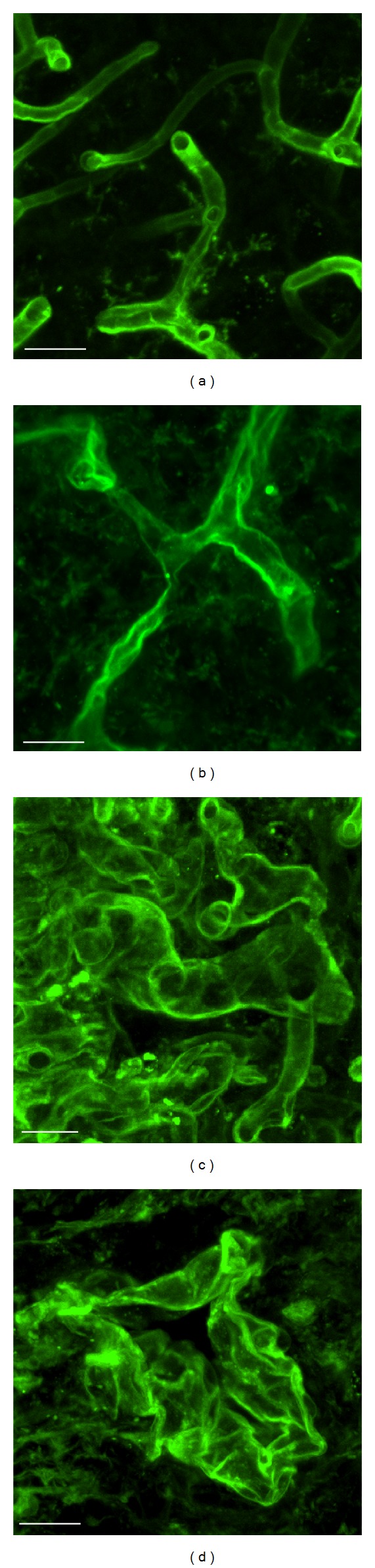
Confocal microphotographs of the microvascular network during development of an ENU-induced glioma. Histochemistry for tomato lectin (LEA-FITC) performed on 40 *μ*m sections. (a) Similar angioarchitecture to the normal brain during the early stage. (b) Tortuous and dilated vessels during the intermediate development stage. (c) The microvascular network is mainly composed of glomeruloid vessels in the periphery area, (d) Occasional huge dilated vessels in the intratumour region (bar scale of 20 *μ*m).

Prior to angiogenesis and to the acquisition of the tumour stem cell phenotype, events like microvascular aberration and subsequent intratumour hypoxia take place. The tumour microvascular network suffers some adaptations to the current requirements of the tumour. The ENU-model allows microvascular changes to be followed before angiogenesis. The adaptive changes of the microvasculature from nonangiogenic gliomas (microtumours) to angiogenic gliomas (macrotumours) involve an increase in the tumour area occupied by the vascular network and a decrease in vascular density [73]. We have demonstrated a transition from the homogeneous capillary network of early stages to an anarchic angioarchitecture in advanced stages (Figure 2). It was found that the vessel density decreased and the vascular size increased with increased malignancy [70]. ENU-gliomas in the initial stage show microvessels very similar to normal brain capillaries (Figure 2(a)), in the intermediate stage they are already tortuous, disorganized, and dilated (Figure 2(b)), and in the advanced stage they become anarchic and aberrant with topographies such as multilayered “glomeruloid tufts”, “garlands” of proliferated vessels (Figure 2(c)) and huge dilated vessels (Figure 2(d)) [70].

Microvascular adaptations in early development stages were based on vasodilatation, endothelium elongation, and permeability increase mediated by VEGF without BBB dysfunction [70]. In malignant gliomas the microvascular adaptations vary according to blood flow perfusion. Permeability increase in intratumour vessels is not enough to supply the metabolic demand, and triggering of the angiogenesis process on the tumour border is necessary.

## 7. The Angiogenic Switch

**Figure 3:**
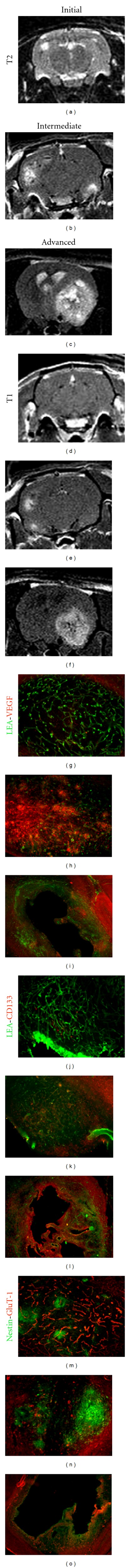
Expression of stem cell markers: CD133 (red) and Nestin (green), proangiogenic factor VEGF165 (red) and microvasculature markers: LEA lectin (green) and GluT-1 (red) during the ENU-glioma development. (a–f) MRI on T2-w and T1-w after gadolinium injection. (a, d) During the initial stage, the ENU-glioma grows in association with the subcortical white matter. It shows a homogeneous hyperintense signal on T2-w (a) and an isointense signal on T1-w (d). (b) ENU-intermediate stage corresponds with the “angiogenic switch.” (c, f) ENU-Glioblastoma displays heterogeneous hyperintense signal on T2 and on T1-w. (g-h) Overexpression of VEGF165 is shown in the intermediate (h) and advanced stages. (i) Also shown in perivascular cells of glomeruloid vessels. (j–l) CD133 expression is found following the intermediate stage (k). (l) ENU-GBM displays plenty of CD133+ cells in the intratumour hypoxic area and bordering the tumour. (m–o) Nestin+ cells are detected in every ENU development stage. Nestin+ cells are grouped into intratumour niches and around the microvessels. (Immunofluorescence images at 100x amplification, except I, l, and o at 40x).

The transition from a prevascular proliferative node to a highly vascularised and progressively outgrowing tumour is referred to as the “angiogenic switch” [74]. Angiogenesis is activated in growing gliomas when the proangiogenic stimuli outweigh the antiangiogenic stimuli. This moment is detected in the ENU-glioma intermediate stage by the presence of overexpression of VEGF and eNOS [75] (Figure 3). In this phase, hypoxia increases VEGF and eNOS expression through the activation of the PI-K/Akt cascade [76, 77].

**Figure 4:**
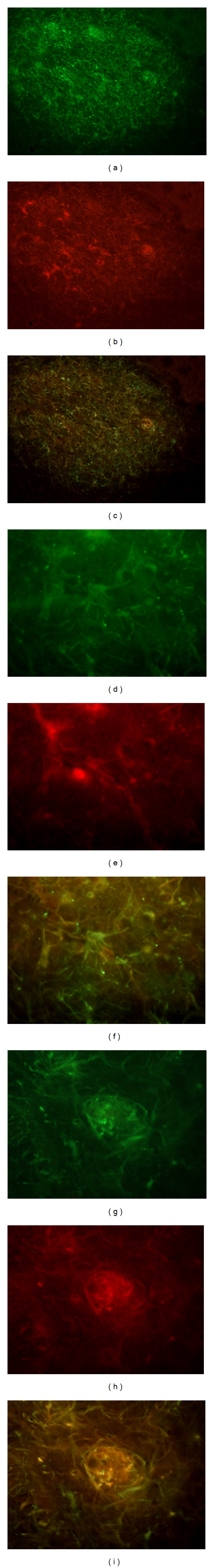
Intermediate ENU glioma stage showing the expression of Nestin (green) and CD133 (red) by double immunofluorescence. (a–c) Intratumour niches of glioma stem cells positive for Nestin (a) and CD133 (b). (c) Both stem cell markers, Nestin and CD133 are coexpressed in some of these cells. (d–f) Isolated cells in the border of the tumour with a shape similar to astrocytes. Although the majority of these cells are Nestin-CD133 positive, there is a greater density of Nestin+ cells than CD133+ ones. (g–i) Perivascular niche of glioma stem cells displaying colocation of both markers.

In this intermediate stage, we identified glioma stem cells by CD133 and Nestin immunostaining (Figure 4). We found that some cells coexpress the Nestin, CD133 and VEGF165 antibodies. These cells were distributed in intratumour niches and perivascular niches around the tortuous and aberrant vessels (intermediate-advanced stages). The distribution of CD133+ cells corresponded mainly to overexpression of VEGF in neoangiogenic border areas and intratumour hypoxic areas of neoplasia [17, 70, 78]. It has been reported that tumour stem cells over-express VEGF, so this cell population could be involved in the process of angiogenesis. Our results agree with the staining of CD133 described in the advanced and intermediate stages of human gliomas. Therefore, CD133 expression has been related to poor prognosis [79].

In the ENU model, as in human GBMs, the angiogenic factor VEGF-A and HIF-1*α* have been immunohistochemically identified in cells located inside the pseudopalisade area around necrosis. This feature provides a link between hypoxia and angiogenesis in malignant gliomas [13, 39] (unpublished data). In addition, we found Nestin+ and CD133+ cells around necrotic areas as well [78]. These findings support the idea that intratumour hypoxia contributes significantly to the selection of glioma stem cells (GSCs) and furthermore these groups of cells could be responsible for the infiltrative process and neoplastic recurrences [9, 56].

## 8. Neovasculature and Tumour Invasiveness

Tumour microvasculature generates specific microenvironments that promote the formation and/or maintenance of brain GSCs. New vessels often show immature walls that are inherently leaky. Tissue may become oedematous. In previous studies, the neoangiogenic area of these tumours was replete with small vessels overexpressing VEGF and displaying an irregular staining for BBB markers [70]. It is a fact that, even though brain tumour vessels appear more “leaky” than their normal tissue counterpart, at least some elements of the BBB in glioblastoma remain intact. Permeability is varied, and the heterogeneity of permeability contributes to uneven distribution of transport products, such us oxygen and chemotherapeutic drugs within the tumour [80].

It has also been proposed that the abnormal blood vessels in gliomas create a vascular niche that houses glioma stem cells [81, 82]. This “perivascular niche” consists of a rich extracellular matrix (ECM) and nontumoural cells, which provide a physical support for tissue architecture. There is consistent evidence about the migration of GSCs using the ECM [83, 84]. If the recruitment of aberrant vascular niches is a critical component in the progression of brain tumours, this might explain why the most aggressive brain tumours are highly angiogenic [19, 85]. Moreover, VEGF isoforms are at least partially bound to the ECM, and their degradation could provide an extra source of growth factors [86].

Calabrese and colleagues [81] showed that endothelial cells (ECs) interact closely with brain tumour stem cells in the perivascular location (termed the perivascular niche) and secrete factors that maintain these cells in a stem cell-like state. Moreover, recent studies of orthotopic glioblastoma xenografts suggested that GSCs of these brain tumours secrete angiogenic factors that promote the recruitment and formation of tumour blood vessels [24].

Folkins and colleagues [60] compared the angiogenesis in tumour xenografts from C6 glioma cells containing either a low or a high fraction of GSCs and found that GSC-high xenograft tumours demonstrated an increased microvessel density and blood perfusion.

## 9. Conclusion

Glioma survival and growth is dependant on angiogenesis. Angiogenesis is a complex process in which several cellular and molecular pathways are involved. Among the cellular pathways, glioma stem cells and vascular endothelial cells play a relevant role, and among the molecular pathways, hypoxia inducible factor -1 (HIF-1) and vascular endothelial growth factor (VEGF) are the most significant. Both molecular mediators are related to glioma stem cells. Therefore, hypoxia selects the GSCs that induce the start of the angiogenesis process by the synthesis of proangiogenic factors such a angiopoietin, ECM factors and VEGF, and decrease angiostatic factors [87]. On the other hand, GSCs mediate vascular adaptation not only by the synthesis of vascular permeability factors or angiogenic factors, but also by differentiation in endothelial cells of the neovascular network.
